# Molecular Dynamics Study on the Effect of Surface Films on the Nanometric Grinding Mechanism of Single-Crystal Silicon

**DOI:** 10.3390/mi16101141

**Published:** 2025-10-02

**Authors:** Meng Li, Di Chang, Pengyue Zhao, Jiubin Tan

**Affiliations:** 1 Center of Ultra-Precision Optoelectronic Instrumentation Engineering, Harbin Institute of Technology, Harbin 150001, China; 25b901080@stu.hit.edu.cn (M.L.); pyzhao@hit.edu.cn (P.Z.); jbtan@hit.edu.cn (J.T.); 2 Key Laboratory of Ultra-Precision Intelligent Instrumentation, Ministry of Industry Information Technology, Harbin 150080, China

**Keywords:** nanogrinding, surface damage, surface films, single-crystal silicon, molecular dynamics

## Abstract

To investigate the influence of surface films on the material removal mechanism of single-crystal silicon during nanogrinding, molecular dynamics (MD) simulations were performed under different surface-film conditions. The simulations examined atomic displacements, grinding forces, radial distribution functions (RDF), phase transformations, temperature distributions, and residual stress distributions to elucidate the damage mechanisms at the surface and subsurface on the nanoscale. In this study, boron nitride (BN) and graphene films were applied to the surface of single-crystal silicon workpieces for nanogrinding simulations. The results reveal that both BN and graphene films effectively suppress chip formation, thereby improving the surface quality of the workpiece, with graphene showing a stronger inhibitory effect on atomic displacements. Both films reduce tangential forces and mitigate grinding force fluctuations, while increasing normal forces; the increase in normal force is smaller with BN. Although both films enlarge the subsurface damage layer (SDL) thickness and exhibit limited suppression of crystalline phase transformations, they help to alleviate surface stress release. In addition, the films reduce the surface and subsurface temperatures, with graphene yielding a lower temperature. Residual stresses beneath the abrasive grain are also reduced when either film is applied. Overall, BN and graphene films can enhance the machined surface quality, but further optimization is required to minimize subsurface damage (SSD), providing useful insights for the optimization of single-crystal silicon nanogrinding processes.

## 1. Introduction

Owing to its excellent mechanical properties and good electrical conductivity, single-crystal silicon (Si) is widely used in semiconductors, photovoltaics, micro-electromechanical systems (MEMS), and precision optics [1,2,3,4]. However, its brittle nature makes it highly susceptible to surface defects and subsurface damage (SSD) during machining. Conventional grinding processes often lead to increased surface roughness and the formation of micro-defects, which degrade device performance and reduce service life, failing to meet high-precision requirements [5,6,7]. To overcome these limitations, nanogrinding techniques have been developed to achieve nanoscale material removal and improved surface flatness through precise control of process parameters, thereby effectively enhancing surface quality [8,9,10]. Nevertheless, relying solely on process parameter optimization remains insufficient to prevent high grinding forces, localized temperature rise, and SSD during nanogrinding, which can compromise material removal stability and machining accuracy. In recent years, the introduction of functional films on workpiece surfaces has been considered an effective approach to improve interfacial friction and thermal conduction, potentially reducing damage, suppressing phase transformations, and enhancing surface quality [11,12,13]. Therefore, it is of great significance to investigate the effects of surface films on the material removal mechanisms of Si during nanogrinding, as this knowledge is crucial for achieving low-damage and ultra-precision machining.

To date, many researchers have investigated the material removal mechanisms of Si during nanogrinding through experiments and simulations. Experimental studies mainly rely on microscopic characterization and mechanical testing to reveal the surface morphology and SSD characteristics during Si nanogrinding. Zhou et al. [14] employed Transmission Electron Microscopy (TEM) to observe SSD and proposed an analysis method based on wafer deformation curvature to investigate the stress distribution of wear-induced damage in Si. Yang et al. [15] experimentally studied the effects of grinding parameters and crystal orientation on SSD in Si, and proposed an anisotropic SSD model validated through Magnetorheological Finishing (MRF) pitting techniques. Wang et al. [16] investigated Si nanomilling based on Atomic Force Microscopy (AFM) probes, established a theoretical model for predicting the machining depth, and revealed the material removal mechanisms, brittle-to-ductile transition, and SSD. Gao et al. [17] studied wafer warpage during ultra-precision Si grinding with back-thinning, and developed a mathematical model linking warpage to machining stress, wafer thickness, subsurface damage layer (SDL) thickness, and Si mechanical properties, which was subsequently validated. However, the complex interactions between abrasive grains and Si during nanogrinding are difficult to explain using conventional theories, and experimental methods cannot easily observe the material removal process in real time.

Simulation studies include first-principles methods, Finite Element Method (FEM), and molecular dynamics (MD). MD can reveal the dynamic material removal mechanisms of nanogrinding at the atomic scale [18,19,20]. Zhang et al. [21] conducted MD simulations of Si nanogrinding at the nanoscale, and investigated structural changes in Si using Common Neighbor Analysis (CNA), radial distribution functions (RDF), and stress distributions. The study revealed that Si lattices undergo compressive and shear deformations, with phase transformation being the primary damage mechanism. Li et al. [22] explored the effects of grinding speed on SSD in Si. The results indicate that the maximum height of grinding chips does not always increase with grinding speed. In addition, grinding forces decrease with increasing grinding speed, which is consistent with the findings of Liu [23] and Li [24]. Huang et al. [25] investigated the effects of different diamond grain shapes on Si nanogrinding. The results show that different grain shapes produce distinct machining outcomes, with cubic-octahedral grains suitable for nanorough grinding and octahedral grains suitable for nanofinishing. Wu et al. [26] employed MD to study the nanogrinding behavior of Si under coupled rotational and translational motion of diamond grains. The study revealed that material removal during Si nanogrinding is governed jointly by root-mean-square (RMS) roughness and grinding depth, and can be classified into three modes: extrusion, plowing, and cutting. In addition, it was found that when the initial RMS roughness is small, the surface smoothness of the workpiece may decrease after a single grinding pass. Chen et al. [27] investigated the atomic-scale mechanisms of vibration-assisted Si grinding. The study revealed that tangential and normal forces fluctuate periodically at twice the vibration frequency. With increasing frequency, tangential/normal forces and amorphous layer thickness decrease, while the scratch area increases.

Existing MD studies have mainly focused on improving Si nanogrinding quality through abrasive grain parameter optimization [28,29,30,31,32], while simulation studies on the effects of surface films on material removal mechanisms remain limited. Dai et al. [33] employed MD to investigate a three-body polishing method of Si under graphene film lubrication. The results show that the graphene film provides superlubrication, reducing both the friction coefficient and atomic removal rate during the three-body polishing process. Furthermore, at a constant polishing depth, lower polishing speeds result in fewer SSD atoms. Wang et al. [34] investigated the role of ductile/brittle (Al/Si) bilayers in Si nanogrinding. The results indicate that the Al-Si interface can impede dislocation motion, significantly enhancing the overall strength of the bilayer structure, and lowering the machining temperature, which contributes to improved wear resistance. Ou et al. [35] employed MD to study the effects of water-film lubrication on 3C-SiC nanogrinding. The study showed that, compared with conventional surfaces, water-film lubrication does not improve the machined surface quality and increases grinding forces and stresses; however, due to the cooling and lubricating effect of water, it can further reduce grinding temperature, potential energy, and SSD depth. The above studies indicate that, in the nanoprocessing of Si or similar hard-brittle materials, the addition of surface films can significantly influence the machining process and outcomes through various mechanisms, effectively improving the ultra-precision machining quality of hard-brittle materials [36,37]. However, the mechanisms by which surface films affect atomic-scale deformation, phase transformation, and SSD evolution in Si remain unclear.

Therefore, this study employed MD to construct Si workpiece models with boron nitride (BN) film, graphene film, and no film, in order to systematically compare nanogrinding behaviors under different surface conditions. Under varying grinding depths and speeds, the effects of these surface conditions on atomic displacement, grinding forces, RDF, phase transformation, temperature distribution, and residual stress during nanogrinding were analyzed. This study helps to reveal, from an atomic perspective, the regulatory role of surface films in Si nanogrinding mechanisms.

## 2. Materials and Methods

### 2.1. Simulation Models

**Figure 1:**
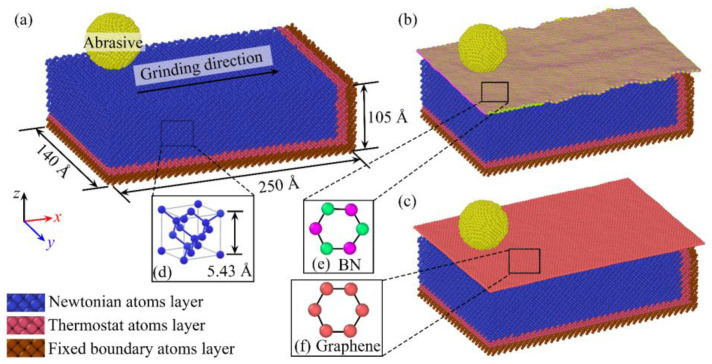
MD simulation models of nanometric grinding: (**a**) Si; (**b**) Si with BN; (**c**) Si with graphene; (**d**) crystalline structure model of single-crystal silicon; (**e**) atomic structure model of BN, and (**f**) atomic structure model of graphene.

To investigate the effect of different surface films on the material removal mechanism of monocrystalline silicon during nano-grinding, this study constructed a MD simulation model based on actual grinding conditions. Figure 1 presents the three MD simulation models for nano-grinding established in this research. The overall structure of each model consists of a Si workpiece, a diamond abrasive grain, and a film layer covering the workpiece surface, where the films are BN and graphene, respectively. In addition, the figure also provides the crystal structure model of Si, as well as the atomic models of BN and graphene. For the sake of consistency in expression, throughout the subsequent text of this paper, the Si model without a film is denoted as Si, the model coated with the BN film is denoted as “Si with BN”, and the model coated with the graphene film is denoted as “Si with Graphene”.

As shown in Figure 1a, the MD simulation model for the nano-grinding process without a film is presented. This model comprises a Si substrate and a diamond abrasive grain with a radius of 4 nm. The diamond abrasive grain contains 47,140 atoms and has a lattice constant of 3.57 Å. The abrasive grain was modeled as a sphere to simplify the geometry and ensure isotropic contact with the workpiece, which is a common assumption in MD nanogrinding studies to capture fundamental mechanisms without the complexity of irregular grain shapes. The Si workpiece has dimensions of 25.0 nm × 14.0 nm × 10.5 nm, a lattice constant of 5.43 Å, and contains approximately 183,752 atoms. The crystal structure of the Si workpiece is displayed in Figure 1d, with its crystal orientations along the *x*-[100], *y*-[010], and *z*-[001] directions, respectively. The workpiece is divided into three atomic layers: the Newtonian atomic layer, the thermostatic atomic layer, and the fixed-boundary atomic layer. In the MD simulation, atoms in the Newtonian atomic layer obey Newton’s second law, and their positions are calculated via integration using the Velocity Verlet algorithm [38]; the Berendsen thermostat method is employed to control the temperature of the thermostatic atomic layer [39]; atoms in the fixed-boundary atomic layer remain stationary at all times. The nano-grinding simulation is conducted on the upper surface of the workpiece along the *x*-direction. Non-periodic boundary conditions (NPBC) are set in the *x*- and *z*-directions, while periodic boundary conditions (PBC) are applied in the *y*-direction. The diamond abrasive grain is treated as a rigid body, with the relative positions of its internal atoms kept fixed, thereby ensuring that only the material response of the Si workpiece is focused on during the simulation. To control the motion of the abrasive grain, a translational velocity was prescribed to all its atoms, while rigid-body rotation was not considered. The grinding depth was realized by first moving the abrasive grain downward along the *z*-direction until the desired penetration was reached, after which the abrasive grain was translated along the *x*-direction to perform nano-grinding. The penetration depth was defined relative to the initial top surface of the workpiece.

As shown in Figure 1b,c, the workpiece surface is coated with BN and graphene films, respectively. Their atomic structures, presented in Figure 1e,f, both exhibit a honeycomb lattice structure with typical layered characteristics. Specifically, graphene, based on a single-layer sp^2^-conjugated carbon network and weak interlayer van der Waals interactions, demonstrates significant superlubricity [40,41,42]; BN, owing to its layered structure, possesses a low friction coefficient [43,44]. In the MD model, the BN and graphene films were bonded to the Si substrate through the interatomic potentials describing their interactions. The initial configurations were generated by placing the film in close contact with the Si surface, after which energy minimization and relaxation were performed to allow the system to reach a stable interface structure. Both BN and graphene films were modeled as a single atomic layer in thickness. These two films differ in structural commonalities and chemical bond properties: the C-C bond is a nonpolar covalent bond, while the B-N bond is a polar covalent bond that generates a dipole moment. This difference leads to variations in interlayer interaction strength, resulting in distinct mechanisms for reducing interface friction and suppressing damage.

During the simulation, the Large-scale Atomic/Molecular Massively Parallel Simulator (LAMMPS) [45] was employed for MD simulations to output atomic trajectories and relevant physical quantities, while the Open Visualization Tool (OVITO) [46] was used for visual analysis of the results. The entire MD simulation of nano-grinding was divided into three stages: initial relaxation, nano-grinding, and final relaxation. First, the conjugate gradient method was applied for energy minimization, followed by a 100 ps relaxation under the NVT ensemble to obtain a stable initial structure. The grinding stage was conducted under the NVE ensemble, where simulations were performed with different grinding speeds (50, 100, and 200 m/s) and grinding depths (1–4 nm) under various surface conditions. Finally, an additional 100 ps relaxation was carried out under the NVT ensemble to ensure the system reached a stable state, enabling the acquisition of the evolution of atomic structures and stress distributions on the surface and subsurface of the specimen. All simulations were repeated under different initial conditions to ensure the reliability and reproducibility of the results.

### 2.2. Potential Functions

To investigate the effect of surface films on the material removal mechanism of Si during nano-grinding, selecting an appropriate potential function is crucial for accurately simulating interatomic interactions. In this study, a hybrid potential function was adopted to describe interatomic interaction relationships, including the Tersoff potential, Lennard-Jones (L-J) potential, and AIREBO potential. The Tersoff potential was used to characterize the interactions between atoms within the Si workpiece and BN film, i.e., Si-Si, B-B, N-N, and B-N interactions. The Tersoff potential can more effectively describe covalently bonded materials [47], and its expression is given by Equation (1).

(1) $$F=\frac{1}{2}\sum_{i}\sum_{i\neq j}E_{ij}$$

where $E_{ij}$ represents the potential energy between atoms *i* and *j*, and its calculation formula is given by Equation (2).

(2) $$E_{ij}=f_{C}(r_{ij})[f_{R}(r_{ij})+b_{ij}f_{A}(r_{ij})]$$

where $f_{C}$ is a smooth cutoff function, which decreases from 1 to 0; $f_{R}$ is the two-body repulsive interaction function between atoms *i* and *j*; $f_{A}$ is the three-body attractive interaction function between atoms *i* and *j*.

In the graphene film, the interatomic interaction (C-C) is described using the AIREBO potential [48]. The AIREBO potential consists of three components: the short-range REBO term, the long-range Lennard-Jones term, and the dihedral torsion term. It can more accurately reflect the structural characteristics of sp^2^ carbon atoms in graphene, and its expression is given by Equation (3).

(3) $$E_{AIREBO}=\sum_{i}\sum_{j>i}[E_{ij}^{REBO}+E_{ij}^{LJ}+\sum_{k\neq i,j}\sum_{l\neq i,j,k}E_{ijkl}^{torsion}]$$

where the REBO term describes short-range covalent bonding interactions, and its expression is given by Equation (4); the L-J term accounts for long-range van der Waals interactions, with its expression as shown in Equation (6); while the torsion term considers the dihedral angle potential during carbon chain rotation, and its expression is presented in Equation (5).

(4) $$E_{ij}^{REBO}=f_{C}\left(r_{ij}\right)\left[V^{R}\left(r_{ij}\right)-b_{ij}V^{A}\left(r_{ij}\right)\right]$$

where $f_{C}\left(r_{ij}\right)$ is a cutoff function, ensuring that interactions smoothly vanish beyond a certain distance; $V^{R}\left(r_{ij}\right)$ is the repulsive potential term; $V^{A}\left(r_{ij}\right)$ is the attractive potential term; $b_{ij}$ is the bond order function, which depends on the arrangement of neighboring atoms and reflects the influence of bond length, bond angle, and local coordination number on bond strength.

(5) $$E_{ijkl}^{torsion}=f_{T}\left(\theta_{ijkl}\right)\cdot \left[1+\cos\left(n\theta_{ijkl}\right)\right]$$

where $\theta_{ijkl}$ is the torsion angle; $f_{T}\left(\theta \right)$ is a regulating function that controls the contribution of the dihedral angle potential; *n* is a periodic parameter that governs the periodic variation in potential energy with angle.

The L-J potential is used to describe the interactions between diamond abrasive atoms and atoms of the Si workpiece, BN, and graphene, i.e., C-Si, C-B, C-N, and C-C interactions. The L-J potential is also applied to characterize the interactions between film atoms and Si workpiece atoms, i.e., B-Si, N-Si, and C-Si interactions. The expression of the L-J potential is given by Equation (6) [49].

(6) $$E(r)=4\varepsilon [{\left(\frac{\sigma }{r}\right)}^{12}-{\left(\frac{\sigma }{r}\right)}^{6}],r<r_{0}$$

where *r* is the distance between two atoms; *σ* and *ε* are the equilibrium distance and cohesive energy, respectively. The L-J potential is also an empirical interatomic potential suitable for multi-component systems, which handles heteronuclear bonds by interpolating the potentials of respective elements. The L-J potential parameters for interactions between various atoms are listed in Table 1. The cutoff radius in this simulation is set to 3 Å to ensure computational efficiency and accuracy [3,23].

### 2.3. Simulation Parameters

During the nano-grinding process of Si, the grinding force of the workpiece can be obtained by differentiating the atomic potential energy, and its expression is given by Equation (7).

(7) $$F_{r}^{ij}=-\frac{\partial E_{ij}}{\partial r_{i}}=4\varepsilon [12(\sigma^{12}/r^{13})-6(\sigma^{6}/r^{7})]$$

where $F_{r}^{ij}$ is the force exerted by atom *j* on atom *i* along the *r*-direction; $E_{ij}$ is the atomic potential energy. The total force acting on atom *i* is obtained by summing the resultant forces from other atoms, as shown in Equation (8).

(8) $$F_{r}^{i}=\sum_{j=1,j\neq i}^{N}F_{r}^{ij}$$

CNA is used to identify crystal structures and obtain their distribution [50]. During the nano-grinding process, the workpiece temperature is calculated from the system kinetic energy, and its calculation formula is given by Equation (9) [51].

(9) $$E_{k}=3/2\cdot NkT$$

where $E_{k}$ is the system kinetic energy; *N* is the number of atoms; *k* is the Boltzmann constant with a value of 1.381 × 10^−23^ J/K; *T* is the system temperature. To analyze the internal stress distribution of the workpiece, the atomic von Mises stress is calculated, and its calculation formula is given by Equation (10).

(10) $$\begin{array}{l} \sigma_{vm}\left(i\right)=\frac{1}{2}\left[{\left(\sigma_{xx}\left(i\right)-\sigma_{yy}\left(i\right)\right)}^{2}+\right.{\left(\sigma_{yy}\left(i\right)-\sigma_{zz}\left(i\right)\right)}^{2}+{\left(\sigma_{xx}\left(i\right)-\sigma_{zz}\left(i\right)\right)}^{2}\\ \;\;\;\;{\left.+6\left(\sigma_{xy}^{2}\left(i\right)+\sigma_{yz}^{2}\left(i\right)+\sigma_{zx}^{2}\left(i\right)\right)\right]}^{\frac{1}{2}} \end{array}$$

where $\sigma_{vm}$ is the von Mises stress of atom *i*; $\sigma_{xx(y,z)}$ are the stress tensor components. The specific parameters used in the MD simulation are listed in Table 2.

## 3. Results and Discussion

### 3.1. Surface Generation Mechanism

**Figure 2:**
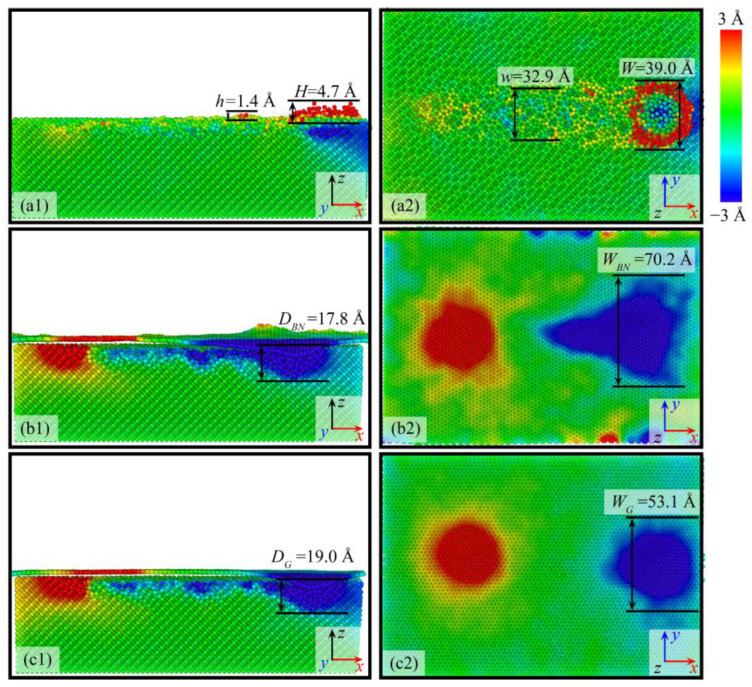
*z*-direction displacement of workpiece atoms under different surface coating conditions: (**a1**,**a2**) Si; (**b1**,**b2**) Si with BN; (**c1**,**c2**) Si with graphene.

As shown in Figure 2, it presents the displacement of atoms along the *z*-axis during the grinding process under different workpiece surface film conditions at an initial temperature of 300 K. Among them, red atoms represent those moving in the positive *z*-axis direction, while blue atoms represent those moving in the negative *z*-axis direction. To facilitate the observation of the displacement of atoms on the workpiece surface, the grinding tool in the figure is hidden. As displayed in Figure 2(a1,a2), when there is no film on the workpiece surface, it can be observed that atomic displacement is mainly concentrated on the front side and bottom of the grinding tool, as well as inside and on both sides of the groove formed by grinding. Under the extrusion of the grinding tool, a part of the atoms on the workpiece surface separates from the workpiece and forms chips. Another part of the atoms moves to the bottom of the grinding tool to form a machined surface, which is consistent with previous experimental results [52]. As the grinding distance gradually increases, the chips accumulated in front of the grinding tool increase, and the chip height gradually rises. Some chips remain on the workpiece surface and are distributed on both sides of the grinding groove. When there is no film on the workpiece surface, the maximum height *H* of the chips is 4.7 Å, and the maximum width *W* of the chips is 39.0 Å. In addition, the pile-up height *h* is 1.4 Å, and the pile-up width *w* is 32.9 Å.

**Figure 3:**
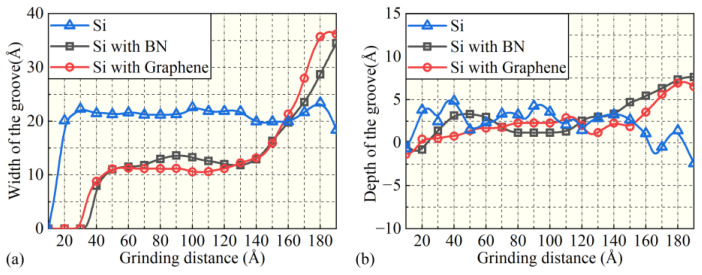
Variation in groove width (**a**) and depth (**b**) on the workpiece surface with grinding distance.

In contrast, for workpieces with BN (Figure 2(b1,b2)) and graphene (Figure 2(c1,c2)) as surface films, the grinding process exhibits atomic displacement characteristics different from those without a film. Under the BN film condition, the grinding action mainly drives atoms to migrate in the negative *z*-axis direction, and no chip separation or pile-up phenomena are observed. Instead, a groove is formed, whose depth and width expand as the grinding distance increases, as shown in Figure 3. For the groove corresponding to the BN film, the maximum depth *D_BN_* = 17.8 Å and the maximum width *W_BN_* = 70.2 Å. This difference stems from the layered crystal structure and excellent mechanical properties of the BN film. The strong bonding interaction between BN atoms and the interaction between the film and the workpiece surface make it easier for grinding stress to drive atoms to squeeze into the matrix interior rather than separate into chips [53].

Under the condition of the graphene film (Figure 2(c1,c2)), the atomic displacement law is similar to that of the BN film: groove formation is also the main phenomenon, and the depth and width of the groove increase continuously with the increase in grinding distance. Among them, the maximum depth of the groove *D_G_* = 19.0 Å and the maximum width *W_G_* = 53.1 Å. As shown in Figure 3, under the graphene film condition, the variation trend of the groove width and depth with the increase in grinding distance is similar to that of the BN film, showing a gradual increase trend. Relying on its unique two-dimensional lattice structure and excellent in-plane mechanical properties, graphene makes it easier for grinding stress to be dissipated through the coordinated displacement of in-plane atoms, thereby inhibiting atomic separation and chip formation, which is manifested as the gradual expansion of the groove [54]. A comprehensive comparison of the simulation results of the workpiece without a film, with the BN film, and with the graphene film shows that the crystal structure, mechanical properties, and interfacial interaction of the films have a significant regulatory effect on the direction of atomic displacement during the grinding process.

**Figure 4:**
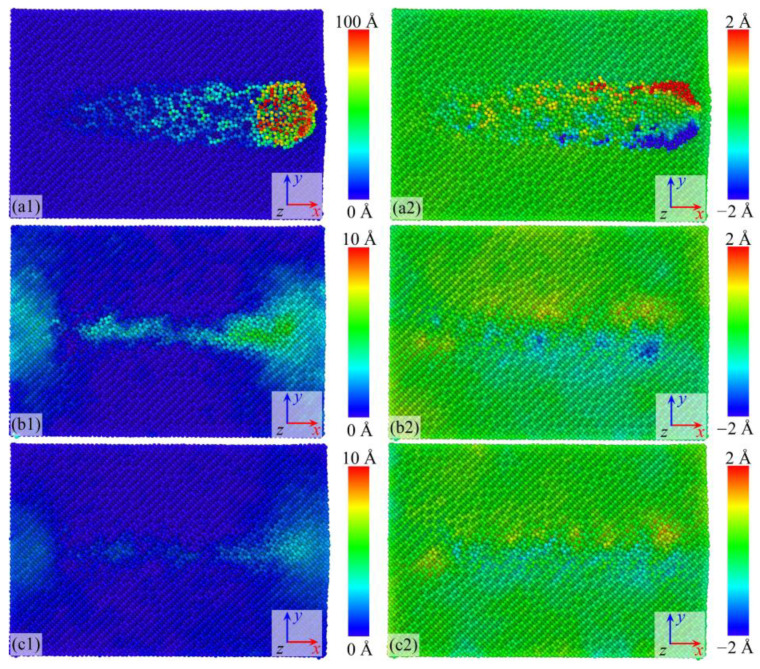
*x* and *y* direction displacements of workpiece atoms under different surface coating conditions: (**a1**,**a2**) Si; (**b1**,**b2**) Si with BN; (**c1**,**c2**) Si with graphene.

Figure 4 shows the atomic displacements of the workpiece in the *x* and *y* directions under three conditions when the nanogrinding distance reaches 20 nm. The atomic displacements exhibit different trends in different directions, reflecting the motion of atoms around the abrasive grain along the grinding direction. As the grinding distance increases, atoms in different regions exhibit distinct movement behaviors. As shown in Figure 4(a1), when no film is applied, the maximum displacement of atoms in the chip region along the *x* direction reaches ~200 Å (red region), while atoms in the groove region move by ~20 Å (light blue region). In comparison, when the film is BN (Figure 4(a2)) or graphene (Figure 4(a3)), atomic displacements along the *x* direction are smaller. With the BN film, the maximum displacement is ~5 Å at the groove bottom, while with the graphene film, the maximum displacement is ~2 Å at the groove bottom. In all three conditions, however, atomic displacements along the *x* direction increase with the grinding distance. In the *y* direction, all three cases exhibit approximately symmetric distributions with respect to the abrasive trajectory, where atoms move toward both the +*y* and −*y* directions with nearly equal numbers on each side. The displacement pattern in the *y* direction is consistent with that in the *x* direction, i.e., increasing with grinding distance, while the smallest *y*-direction displacement occurs with graphene and the largest without a film.

**Figure 5:**
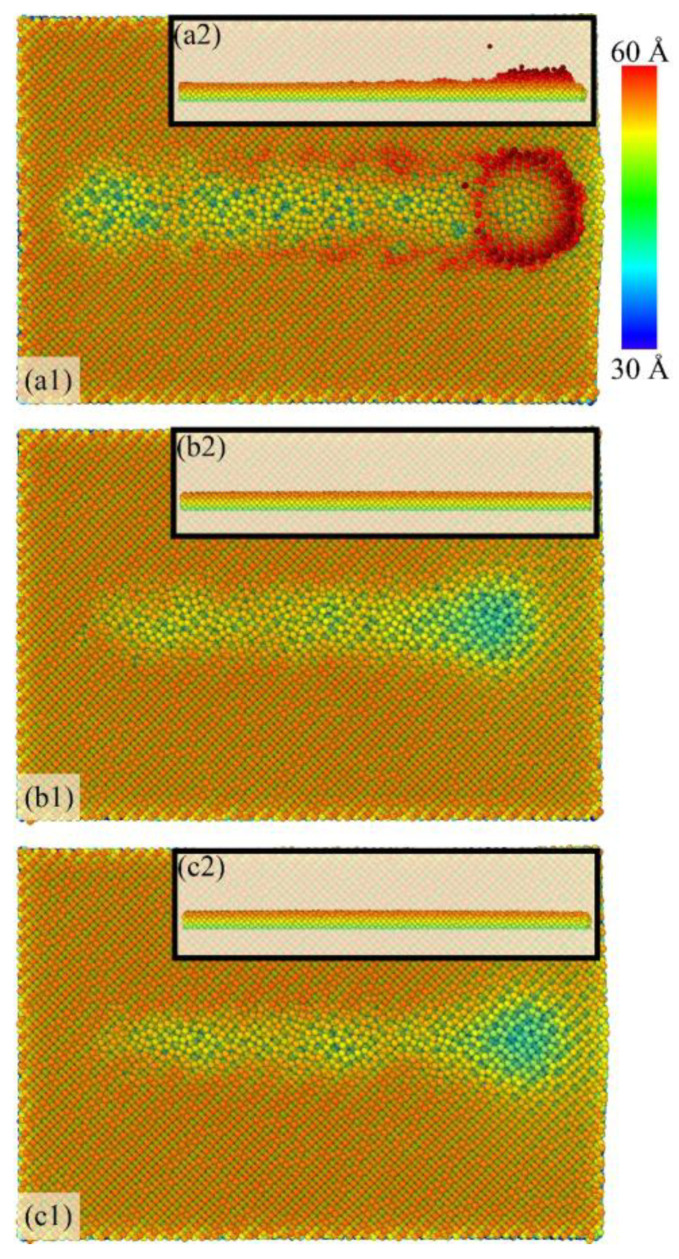
Surface quality of the workpiece under different surface coating conditions: (**a1**,**a2**) Si; (**b1**,**b2**) Si with BN; (**c1**,**c2**) Si with graphene.

During nanogrinding, the surface quality of the workpiece without a film, with a BN film, and with a graphene film is shown in Figure 5. The color bar represents the *z*-coordinate of surface atoms relative to the initial bottom surface of the workpiece, providing an atomic-scale indication of surface morphology and machining quality. Among them, (a1)–(c1) present the surface quality of the workpiece in the *xy*-plane, while (a2)–(c2) show enlarged views of the chips in the *xz*-plane. Under the extrusion of the abrasive tool, some surface atoms form chips in front of the abrasive, while others accumulate at the groove edges on both sides. The height and morphology of the edge pile-up lead to deviations between the actual machined surface and the ideal one; thus, reducing the height of edge pile-up is essential for improving surface quality. As illustrated in Figure 5, introducing BN and graphene films effectively prevents chip formation during nanogrinding and significantly suppresses edge pile-up. Without a film (Figure 5(a1,a2)), extrusion by the abrasive tool causes large-scale atom separation, forming chips (*H* ≈ 4.7 Å). Meanwhile, the edge pile-up at the groove sides is relatively small (*h* ≈ 1.4 Å) but irregular in morphology, which considerably deteriorates surface flatness. When the surface film is BN (Figure 5(b1,b2)) or graphene (Figure 5(c1,c2)), atoms deform more orderly under grinding stress, the edge pile-up height is markedly reduced, and the atomic arrangement of the surface becomes closer to the ideal machined state. In summary, applying surface films on Si during nanogrinding effectively suppresses the formation of chips and edge pile-up, thereby enhancing the surface quality of the workpiece. Both BN and graphene improve machining quality by regulating atomic displacements and suppressing pile-up during the nanogrinding process.

### 3.2. Analysis of Mechanical Properties

**Figure 6:**
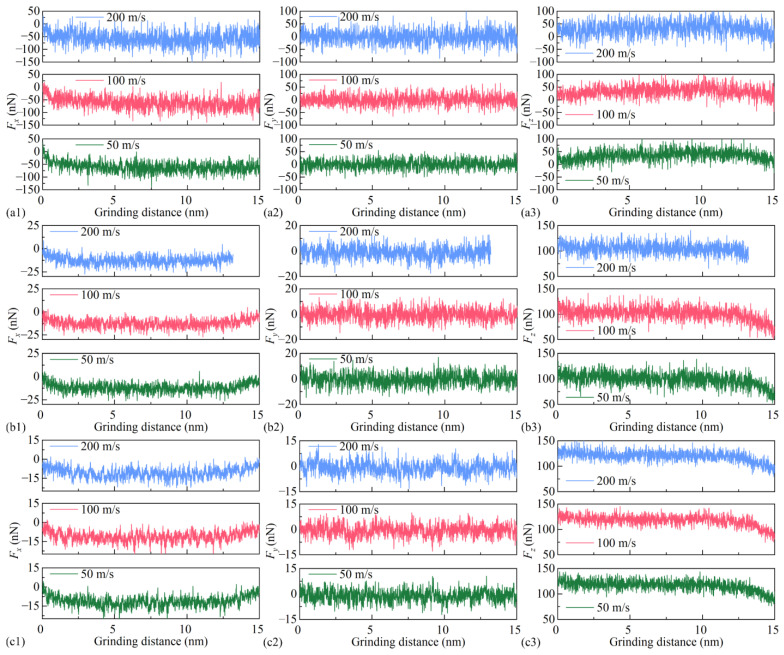
Variation in tangential force (*F_x_*), orthogonal force (*F_y_*), and normal force (*F_z_*) with grinding distance under different surface coating conditions at grinding speeds of 50, 100, and 200 m/s: (**a1**–**a3**) Si; (**b1**–**b3**) Si with BN; (**c1**–**c3**) Si with graphene.

**Figure 7:**
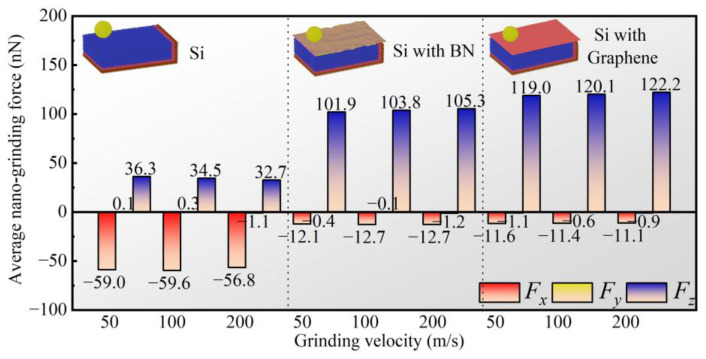
Average tangential (*F_x_*), orthogonal (*F_y_*), and normal (*F_z_*) forces during the nanogrinding process under different grinding speeds and surface coating conditions.

In nanoscale grinding, the grinding force is a critical parameter describing the interaction between the grinding tool and the workpiece, directly affecting the surface quality and the formation of the SDL. Due to differences in surface films, the characteristics of the force components in the *x*, *y*, and *z* directions vary, leading to distinct trends in the grinding forces along these directions. The nanoscale grinding force acting on the workpiece can be orthogonally decomposed into three components: tangential force *F_x_*, lateral force *F_y_*, and normal force *F_z_*. Figure 6 illustrates the variation curves of each force component with increasing grinding distance under different grinding speeds and surface film conditions, while Figure 7 presents the average values of the three components within the grinding distance of 0–15 nm. As shown in Figure 6(a1–c3), *F_x_*, *F_y_*, and *F_z_* all exhibit high-frequency oscillations with large amplitudes. Under all surface conditions, higher grinding speeds result in more intense fluctuations and larger amplitudes of *F_x_*, *F_y_*, and *F_z_*, owing to the shorter contact time and higher impact frequency between abrasive grains and the workpiece. The influence of grinding speed on force is most pronounced without a film, as shown in Figure 6(a1–a3). When covered with BN or graphene, the fluctuation intensity and amplitude of the grinding forces are significantly reduced. This reduction can be attributed to the lower friction coefficient and superlubricity of BN and graphene, which effectively suppress force oscillations, making them suitable for high-speed grinding scenarios. Among the three cases, graphene demonstrates the best suppression of grinding force fluctuations, followed by BN, while the no-film condition performs the worst.

As shown in Figure 7, the average values of *F_x_*, *F_y_*, and *F_z_* during nanoscale grinding are presented under different grinding speeds and surface film conditions. Under the BN film condition, the average tangential forces corresponding to the three grinding speeds are −12.1 nN, −12.7 nN, and −12.7 nN, respectively. Compared with the no-film case, the average tangential force is reduced by 79.49%, 78.69%, and 77.64%, respectively. Under the graphene film condition, the average tangential forces corresponding to the three grinding speeds are −11.6 nN, −11.4 nN, and −11.1 nN, respectively, representing reductions of 80.34%, 80.87%, and 80.46% compared with the no-film case. This reduction can be attributed to the layered structures and extremely low interlayer shear strengths of BN and graphene. They form an easily sheared lubricating film between the abrasive grain and the workpiece, significantly decreasing the friction coefficient. Unlike *F_x_* and *F_z_*, the average value of *F_y_* remains close to zero due to the structural symmetry of the workpiece model. In contrast to the trend observed for the tangential force, the average normal force increases under the film-covered conditions at all grinding speeds. For the BN film, the average normal forces at the three grinding speeds are 101.9 nN, 103.8 nN, and 105.3 nN, corresponding to increases of 180.72%, 200.87%, and 222.02% compared with the no-film case. For the graphene film, the corresponding average normal forces are 119.0 nN, 120.1 nN, and 122.2 nN, which are 227.82%, 248.12%, and 273.70% higher than those of the no-film case. Although the incorporation of BN and graphene reduces friction, it simultaneously alters the effective stiffness at the contact interface between the abrasive grain and the film. Consequently, larger normal forces are required for the abrasive grain to achieve effective material removal or deformation, which is the fundamental reason for the increase in the normal force.

A further analysis highlights the influence of different film materials on grinding forces. The average normal force under the graphene film is consistently higher than that under the BN film. This suggests that graphene exhibits stronger lubricity or that the film generates a different mechanical response, thereby requiring a larger normal force to achieve a material removal state comparable to that under the BN film. In addition, the average tangential force under the graphene film is lower than that under the BN film, confirming that graphene generally provides superior lubricating performance and a lower friction coefficient. Thus, under higher normal forces, smaller tangential forces are realized in the presence of the graphene film.

As shown in Figure 7, when the grinding speed increases from 50 m/s to 200 m/s, the average tangential and normal forces under both film-covered conditions exhibit no significant changes. The variation rates of the average tangential and normal forces remain below 5% as the grinding speed increases. This indicates that the grinding speed has no substantial effect on the grinding forces under either film condition. In contrast, in the absence of a surface film, the grinding forces decrease linearly with increasing grinding speed, suggesting that higher speeds weaken the interaction between the abrasive grain and the workpiece.

**Figure 8:**
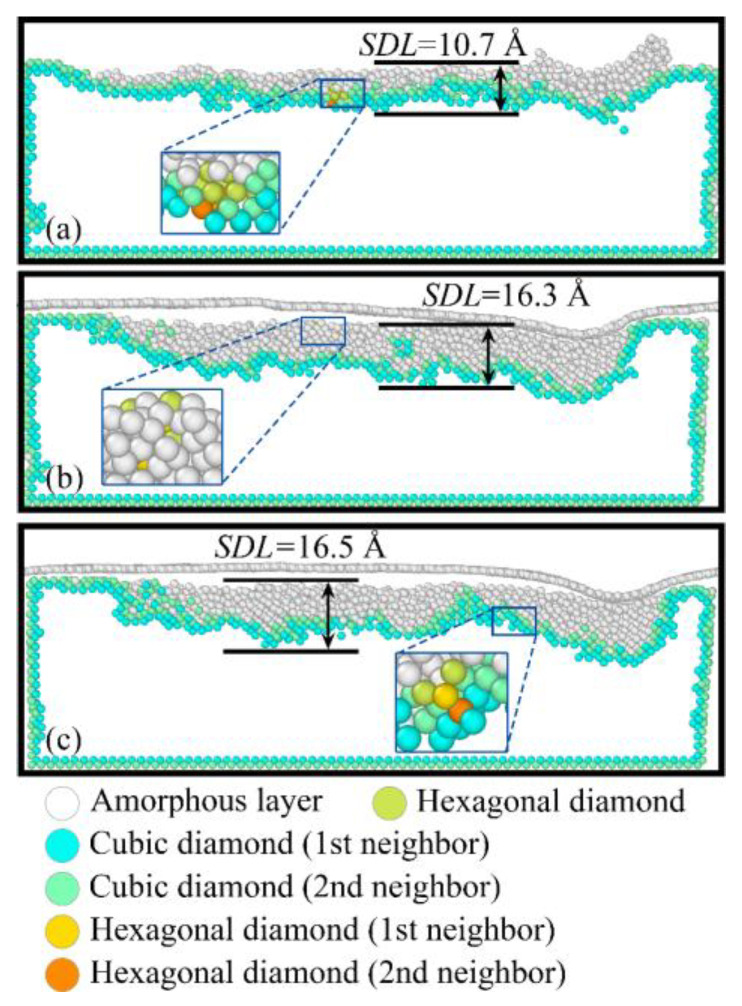
Thickness of the SDL and corresponding zoomed-in views under different surface coating conditions: (**a**) Si; (**b**) Si with BN; (**c**) Si with graphene.

During nanogrinding, intense extrusion and friction between the abrasive grain and the workpiece surface lead to changes in the crystal structure of the surface and subsurface regions, resulting in the formation of a SDL. The size of the SDL has a significant impact on the material’s surface quality and mechanical properties, making it essential to investigate the formation and evolution of SDL in detail. Figure 8 shows the crystal structures of the SDL and corresponding local magnifications under different surface film conditions when the grinding distance reaches 20 nm. The central atoms of cubic and hexagonal crystal structures, along with their first and second nearest neighbors (1st neighbor, 2nd neighbor), are extracted and colored, while other atoms in amorphous structures are displayed in white. To better visualize phase transformation within the SDL, the deeper layers of cubic crystal structure that have not undergone phase change are hidden in Figure 8.

**Figure 9:**
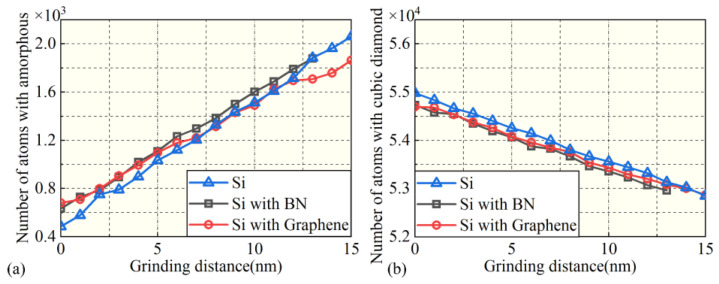
Variation in atomic populations under different surface coating conditions: (**a**) amorphous, (**b**) cubic diamond.

**Figure 10:**
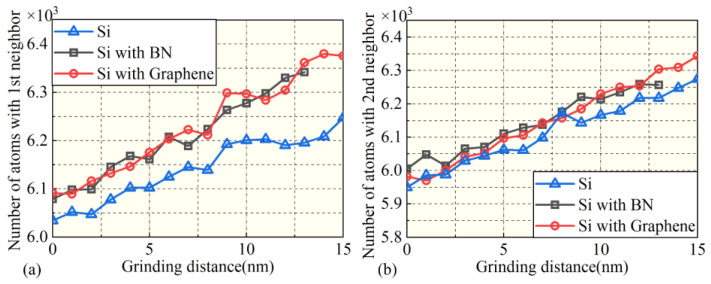
Variation in atomic populations under different surface coating conditions: (**a**) 1st neighbor, (**b**) 2nd neighbor.

Before nanogrinding, the internal atomic structure of the workpiece is cubic diamond, and the surface atomic structure consists of the first and second nearest neighbors of the cubic diamond structure. During nanogrinding, the surface atomic structure transforms from cubic diamond to amorphous. As shown in Figure 9, with increasing grinding distance, the number of atoms in the amorphous structure increases linearly, while the number of cubic diamond atoms decreases linearly. The observed amorphization in the surface and subsurface regions of Si can be attributed to the localized high temperatures induced by the intense interaction between the abrasive grain and the workpiece. Moreover, the edges of the SDL are mainly composed of the first and second nearest neighbors of the cubic diamond structure. Hexagonal crystal structures and their first and second nearest neighbors are observed in the internal regions of the SDL under all three film conditions, as shown in the local magnifications of Figure 8a–c. Figure 10 presents the variation in the number of first and second nearest neighbors in the hexagonal crystal structure with grinding distance under the three surface film conditions. It can be seen that, for all three surface film conditions, the number of first and second nearest neighbors in the hexagonal crystal structure increases with increasing grinding distance.

The thickness of the SDL is used to characterize the extent of the subsurface damage region on the workpiece surface after nanogrinding under different film conditions, defined as the distance between the lowest atom in the SDL and the machined surface of the workpiece. The thickness of the SDL varies with changes in the surface film. Experimental results indicate that adding a surface film to the Si workpiece significantly increases the SDL thickness. Compared with the workpiece without a surface film, the SDL thickness increases from 10.7 Å to 16.3 Å (approximately 52.34% increase) when the film is BN, and from 10.7 Å to 16.5 Å (approximately 54.21% increase) when the film is graphene. These results demonstrate that the addition of surface films on Si enlarges the SDL region, thereby intensifying subsurface damage and reducing the machined surface quality of the workpiece.

### 3.3. Analysis of Temperature Distribution

During nanogrinding, the interaction between the abrasive grain and the workpiece significantly alters the temperature distribution on the workpiece surface, thereby affecting the thickness of the surface layer and phase transformation behavior. Therefore, it is essential to investigate the temperature distribution patterns on both the surface and subsurface regions of the workpiece.

**Figure 11:**
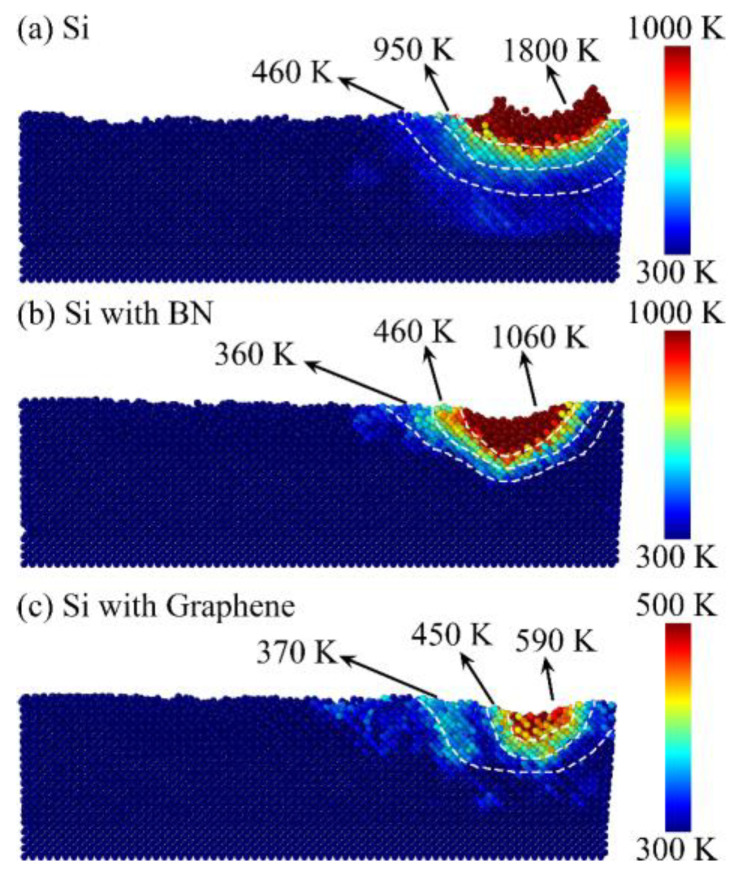
Temperature distribution on the workpiece surface and subsurface under different surface coating conditions during grinding.

To further investigate the temperature distribution on the surface and subsurface of the workpiece under different film conditions at a nanogrinding distance of 20 nm, and to explore the origin of silicon atomic phases, Figure 11 presents the grinding temperature distributions. Intense interactions between the abrasive grain and the workpiece surface lead to a significant increase in temperature in both the surface and subsurface regions. Under the BN film condition, the surface temperature decreases from 1800 K to 1060 K, and the subsurface temperature decreases from 950 K to 460 K. Under the graphene film condition, the surface temperature decreases from 1800 K to 590 K, and the subsurface temperature decreases from 950 K to 450 K. These results indicate that adding a surface film effectively reduces the temperature distribution on both the surface and subsurface, with the surface temperature eventually stabilizing near the initial grinding temperature. Compared with the no-film condition, BN and graphene significantly reduce the friction coefficient between the abrasive grain and the workpiece due to their excellent lubricating properties, thereby lowering the heat generated during grinding. In addition, the high thermal conductivity of the film materials accelerates heat dissipation in the surface and subsurface regions, effectively preventing local heat accumulation. Moreover, during nanogrinding, the film fills surface defects and increases the contact area, allowing heat to be more efficiently transferred into the film, further reducing the surface and subsurface temperatures. A comparison between BN and graphene films shows that graphene, with superior thermal conductivity and lubricating properties, more effectively lowers the temperature distribution in both surface and subsurface regions, thereby mitigating damage to the machined surface and subsurface quality.

### 3.4. Analysis of Phase Transformation

In the study of nanogrinding of Si workpieces, the RDF and coordination number (*CN*) methods are employed to further investigate changes in the atomic crystal structure during nanogrinding, thereby analyzing the phase transformation of silicon atomic structures.

**Figure 12:**
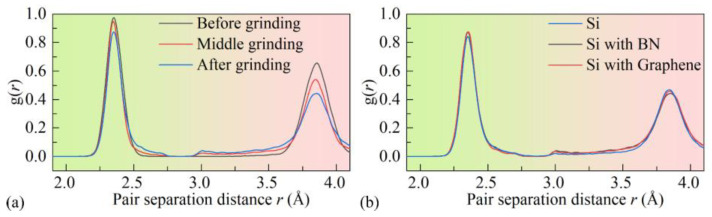
RDF curves of the workpiece at different grinding stages (**a**) and under different surface coating conditions (**b**).

As shown in Figure 12a, the RDF curves at different stages of nanogrinding are presented, where the black curve represents the state before grinding, the red curve corresponds to the process of grinding, and the blue curve indicates the state after grinding. Two main peaks can be observed: the first peak at 2.35 Å corresponds to the distance between first-nearest neighbors in the Si-I phase, while the second peak at 3.83 Å corresponds to the distance between second-nearest neighbors in the Si-I phase. To comprehensively capture this critical structural information, a cutoff radius of 4.0 Å is selected in the RDF analysis to include both first- and second-nearest neighbors. The first-nearest neighbors reflect the direct bonding of silicon atoms, whereas the second-nearest neighbors indicate the order of the local tetrahedral structure; both are crucial for elucidating phase transformations during nanogrinding. In contrast, higher-order neighbors are more susceptible to thermal disturbances and stress effects, often exhibiting blurred and non-representative peaks, and are therefore excluded from the analysis. During nanogrinding, the two peaks of the Si-I phase decrease, indicating that the local order is disrupted and phase transformation occurs. After grinding, the peaks further decline, showing that the damaged crystal structure does not recover under the simulated conditions, with substantial atomic structure disruption persisting. Moreover, the RDF peaks do not return to their pre-grinding heights, suggesting that, within the simulation conditions and timescale of this study, the crystal structure exhibits irreversible phase transformation.

Figure 12b presents the RDF curves of the Si workpiece after nanogrinding under different surface film conditions. It can be observed that, with the addition of surface films, the two main RDF peaks show no significant changes, and the peak heights remain largely stable. This indicates that, from the perspective of RDF, surface films have a limited effect on the stability of the local crystal structure, and their ability to suppress phase transformation or reduce surface/subsurface damage may be negligible.

**Figure 13:**
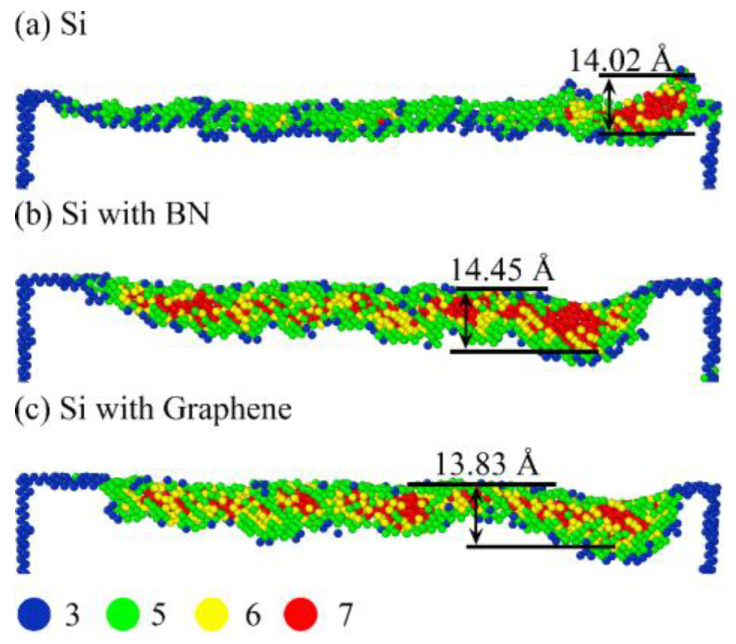
CN distribution during nanogrinding of single-crystal Si under different surface coating conditions: (**a**) Si; (**b**) Si with BN; (**c**) Si with graphene.

To further investigate the structural evolution of the SDL during the phase transformation of crystalline Si (e.g., Si-I to Si-II transition), a detailed atomic analysis was conducted using the *CN* method. In the *CN* analysis, a cutoff radius of 3.2 Å was selected, which is slightly larger than the Si-Si first-neighbor bond length in the Si-I phase (~2.35 Å). This allows for complete counting of first-nearest neighbors while excluding interference from second-nearest neighbors, thereby accurately reflecting direct atomic coordination, consistent with previous molecular dynamics studies on Si [55]. Figure 13a–c show the *CN* distributions in the SDL region of the workpiece under no-film, BN-film, and graphene-film conditions, respectively. *CN* values of atoms in the SDL region are extracted and color-coded, while the abrasive grain and atoms in the standard cubic crystal structure (*CN* = 4) are hidden for clearer visualization. *CN* = 3 (blue) corresponds to low-coordination surface atoms, *CN* = 5 (green) to five-coordinated Si atoms in the body-centered tetragonal structure (bct-5Si), *CN* = 6 (yellow) to six-coordinated atoms in the Si-II phase (β-Si), and *CN* = 7 (red) to seven-coordinated atoms formed under high-pressure conditions. It can be observed that a large number of *CN* = 5 atoms exist at the workpiece surface and SDL edges, whereas *CN* = 6 and *CN* = 7 atoms are mainly distributed within the SDL, with a few *CN* = 3 atoms appearing at the machined surface and SDL bottom. Most *CN* = 7 atoms are concentrated beneath the abrasive grain, due to the maximum pressure exerted by the grain at this region during nanogrinding, resulting in a significant generation of *CN* = 7 atoms. After nanogrinding, the surface pressure is released, and the *CN* distribution of the workpiece without a film differs significantly from those with BN or graphene films. In the machined region, a considerable number of *CN* = 7 atoms remain in the BN- and graphene-coated workpieces, whereas nearly all *CN* = 7 atoms in the uncoated workpiece convert to *CN* = 5 and *CN* = 6. Furthermore, the addition of surface films does not significantly alter the range of the phase transformation region, with the height distributions of *CN* = 7 atoms measured at 14.02 Å, 14.45 Å, and 13.83 Å, respectively. These results suggest that, compared to the complete pressure release in the no-film condition, the layered structures of BN and graphene films may partially retard the pressure release, retaining some *CN* = 7 atoms in the machined region. Comparison of the number of *CN* = 7 atoms beneath the abrasive grain shows the highest count in the no-film case, indicating that the films reduce the pressure in this region and consequently limit *CN* = 7 atom formation. Notably, the addition of surface films moderately affects the degree of phase transformation but does not significantly change the spatial extent of the phase transformation region. This suggests that the primary role of the films may not be to suppress phase transformation in the SDL, but rather to delay the reversal of high-pressure phases.

**Figure 14:**
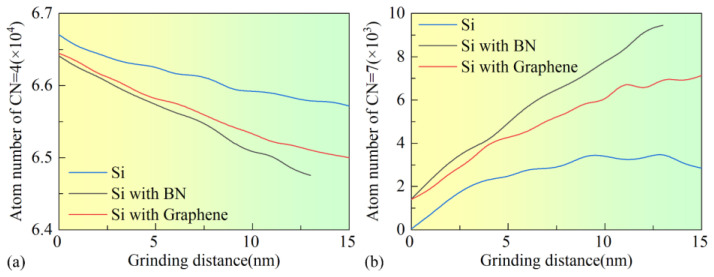
Variation of *CN* in single-crystal Si under different surface coating conditions: (**a**) *CN* = 4, (**b**) *CN* = 7.

Figure 14 presents the variation in *CN* = 4 and *CN* = 7 atom counts with nanogrinding distance for workpieces under different surface film conditions. It can be observed that, in the BN-coated workpiece, the number of *CN* = 4 atoms decreases significantly, while the number of *CN* = 7 atoms exhibits the fastest growth. In comparison, the graphene-coated workpiece shows fewer *CN* = 4 atoms than the uncoated workpiece, while the number of *CN* = 7 atoms is higher. These results indicate that the introduction of surface films may promote the transformation from four-coordinated structures to higher-coordinated structures within the SDL region, thereby intensifying the phase transformation process and affecting the subsurface machining quality. Among the films, BN exhibits the most pronounced effect, with the degree of phase transformation being more significant than in the uncoated and graphene-coated workpieces.

### 3.5. Analysis of Residual Stress

After nanogrinding, the release of residual stress induces elastic recovery of the Si workpiece surface, partially restoring its initial morphology. In addition, extensive phase transformations of silicon atoms occur in the surface and subsurface layers, resulting in the accumulation of residual stress. Therefore, analyzing the distribution of residual stress during nanogrinding is crucial.

**Figure 15:**
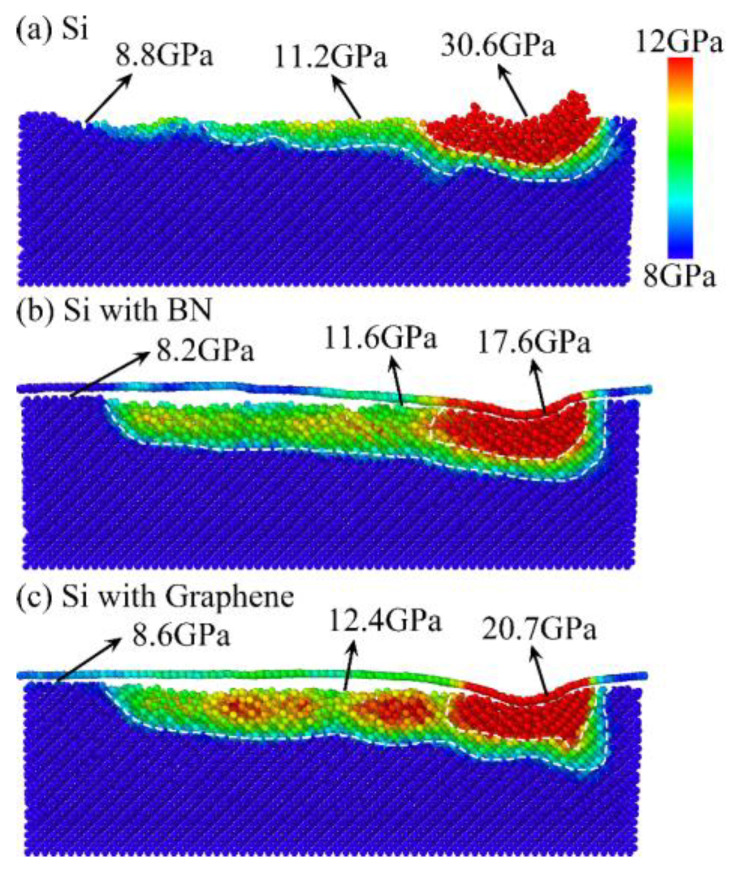
von Mises stress distribution under different surface coating conditions.

Figure 15 presents the von Mises stress distributions under different surface film conditions, with approximate stress regions indicated by color: the red region beneath the abrasive grain, the green transition region, and the blue machined surface region. The maximum von Mises stress is observed in the red region beneath the abrasive grain, followed by the green transition region, while the blue region exhibits the lowest residual stress. This distribution arises from the intense compression between the abrasive grain and the workpiece surface during nanogrinding. It can be seen that, after nanogrinding, the von Mises stress in the workpiece surface (green and blue regions) is significantly reduced due to pressure release and partial phase transformation of silicon atoms, as shown in Figure 13. The maximum von Mises stresses under different surface film conditions are 30.6 GPa for Si, 17.6 GPa for Si with BN, and 20.7 GPa for Si with graphene. The addition of surface films significantly reduces the von Mises stress beneath the abrasive grain (red region), by 42.48% for Si with BN and 32.35% for Si with graphene. However, the von Mises stress on the machined surface (green and blue regions) changes little compared to the uncoated workpiece, with stress values remaining nearly identical. These results indicate that the effect of surface films on von Mises stress is limited; only the stress beneath the abrasive grain is noticeably reduced by the presence of the film.

**Figure 16:**
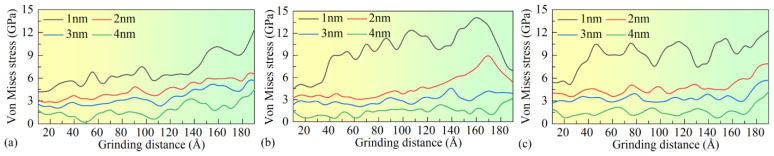
Variation in von Mises stress with grinding distance under different surface coating conditions at grinding depths of 1, 2, 3, and 4 nm: (**a**) Si; (**b**) Si with BN; (**c**) Si with graphene.

To further investigate the von Mises stress distribution in the Si workpiece during nanogrinding, the von Mises stress under different nanogrinding depths and surface film conditions was analyzed. As shown in Figure 16, with the increase in nanogrinding depth, the von Mises stress in the workpiece decreases significantly under all three surface film conditions. When the nanogrinding depth increases from 1 nm to 4 nm, the average von Mises stress in the workpiece decreases from 6.9 GPa (Si), 9.7 GPa (Si with BN), and 9.1 GPa (Si with Graphene) to 1.7 GPa, 1.4 GPa, and 1.6 GPa, respectively. These simulation results indicate that increasing the nanogrinding depth can effectively reduce the von Mises stress within the workpiece.

### 3.6. General Discussion

In summary, the results of Section 3.1, Section 3.2, Section 3.3, Section 3.4 and Section 3.5 reveal a consistent picture of the effects of surface films on the nanogrinding behavior of single-crystal Si. The presence of BN and graphene films alters atomic displacement patterns, suppresses chip formation, reduces edge pile-up, and enhances surface quality. Mechanical analysis shows that these films significantly reduce tangential force fluctuations while slightly increasing normal forces, which is attributed to their excellent lubricating and layered structures. Phase transformation and SDL analyses indicate that surface films influence the degree and distribution of subsurface damage, partially retaining high-pressure phases and affecting local crystal structures. Temperature and residual stress analyses further confirm that surface films mitigate thermal and mechanical damage, with graphene exhibiting superior performance due to its higher thermal conductivity and in-plane mechanical strength. Overall, these findings highlight the crucial role of surface films in regulating atomic-scale material removal, force interactions, thermal response, and phase evolution during nanogrinding. The results provide valuable guidance for optimizing surface film application to improve machining quality and precision in nanoscale grinding processes.

## 4. Conclusions

In this study, MD simulations were performed to systematically investigate the influence of BN and graphene films on the nanogrinding behavior of Si. The main conclusions can be summarized as follows:

(1) Different surface film conditions significantly affect machined surface morphology and subsurface damage. Without films, chip formation and groove-end pile-up dominate, whereas BN and graphene films suppress chip formation and promote groove expansion. Graphene provides stronger constraint on atomic displacements, leading to better surface quality.
(2) Both BN and graphene films reduce the average tangential force by 77.64–80.87% while increasing the average normal force by 180.72–273.70%. Graphene shows a stronger suppression of tangential force but a higher increase in normal force. In addition, graphene effectively reduces force fluctuations, making it more suitable for high-speed nanogrinding. The effect of grinding speed on average forces is minimal under film-covered conditions, whereas without a film, higher speeds reduce the average forces.
(3) The presence of surface films increases the SDL thickness by 52.34% (BN)–54.21% (graphene). However, their ability to inhibit phase transformation is limited. By slowing the release of surface pressure, high-coordination atoms (*CN* = 7) are partially retained, influencing the phase transformation process.
(4) Both BN and graphene films decrease surface and subsurface temperatures, with graphene showing better heat dissipation due to its high thermal conductivity. Both films also reduce von Mises stress beneath the abrasive grain, and increasing the grinding depth further lowers the internal stress.
(5) Uncoated Si workpieces are prone to chip formation and pile-up, favoring high-efficiency material removal. BN films reduce tangential force, friction, and temperature rise, making them suitable for conventional precision nanogrinding. Graphene films, with superior lubrication and heat conduction, perform better in improving surface quality, reducing temperature, and supporting high-speed grinding, but they also lead to higher normal force and more severe subsurface damage.

This work considers only single abrasive grain nanogrinding, while actual grinding involves the coupling effect of multiple abrasive grains. Moreover, the size of the MD workpiece is much smaller than in real experiments, which may affect the accuracy of quantitative results. Although different grinding speeds and depths were examined in the simulations, further experimental validation is necessary to confirm the observed trends. The simulation results could be experimentally validated by performing nanogrinding tests on Si workpieces with BN or graphene films and characterizing the surface and subsurface using AFM or TEM. Future work should explore multi-grain coupling effects and optimize film design to mitigate subsurface damage while achieving higher-quality machining.

## Figures and Tables

**Table 1:** Parameters of the L-J potential describing interatomic interactions.

Element	*σ*(Å)	*ε*(meV)	*r*_0_
C-Si	4.0669	8.9092	3.0
C-B	3.9653	5.9615	3.0
C-N	3.7732	6.0089	3.0
C-C	3.8510	4.5532	3.0
B-Si	4.1876	11.6648	3.0
N-Si	3.9848	11.7575	3.0

**Table 2:** Calculation parameters employed in the molecular dynamics simulations.

Parameters	Value
Specimen materials	Silicon
Dimension of workpiece (nm^3^)	25.0 × 14.0 × 10.5
Surface crystal orientation of workpiece	{100}
Grinding directions	[100]
Material of grinding grit	Diamond
Radius of grinding grit (nm)	4.0
Potential function	Tersoff, L-J, AIREBO
Initial temperature (K)	293
Nano-grinding speed (m/s)	50, 100, 200
Nano-grinding depth (nm)	1, 2, 3, 4
Nano-grinding distance (nm)	20
Timestep (fs)	1

## Data Availability

The data presented in this study are available on request from the corresponding author due to privacy.
